# The Photochemistry of Benzotriazole Derivatives. Part 2: Photolysis of 1-Substituted Benzotriazole Arylhydrazones: New Route to Phenanthridin-6-yl-2-phenyldiazines

**DOI:** 10.3390/molecules161210256

**Published:** 2011-12-08

**Authors:** Nader A. Al-Jalal, Maher R. Ibrahim, Nouria A. Al-Awadi, Mohamed H. Elnagdi

**Affiliations:** Department of Chemistry, Faculty of Science, Kuwait University, P.O. Box 5969, Safat 13060, Kuwait

**Keywords:** 1,2,3-benzotriazole, phenanthridin-6-yl-2-phenyldiazine, phenanthridin-6(5*H*)-one, 1-anilinobenzimidazole, 1*H*-benzimidazole, 2-benzoyl-1*H*-benzoxazole, photolysis

## Abstract

Irradiation of 1-substituted benzotriazole arylhydrazones **3a–c**, **4a**,**b** and **5a**,**b** with a 16 W low pressure mercury arc-lamp (254 nm) for 24 h gave phenanthridin-6-yl-2-phenyldiazines **9a–c**, phenanthridin-6(5*H*)-ones **10a–c**, 1-anilinobenzimidazoles **11a–c**, 2-aryl-1*H*-benzimidazoles **12a–c**, 1-arylamino-1*H-*benzimidazol-2-carboxylic acid ethyl esters **14a**,**b**, 1-aryl-1*H*, 9*H*-benzo [4,5][1,2,3] triazolo[1,2-*a*]tetrazole-3-carboxylic acid ethyl esters **16a**,**b**, 1-arylamino-2-benzoylbenzimidazoles **18a**,**b** and 2-benzoylbenzoxazole **21**.

## 1. Introduction

The behavior of benzotriazole and its derivatives under pyrolytic and photolytic conditions has already received considerable attention. In particular, 1-substituted-1*H*-benzotriazoles pyrolyze via elimination of N_2_ to give a 1,3-diradical intermediate which can interact with aromatic or unsaturated substituents to give cyclic and rearranged products [1,2,3,4,5,6,7,8,9,10]. In the earlier paper in this series, we have reported the photolysis of *N1*-vinylsubstituted benzotriazole derivatives **1** into 2-acyl-3-dimethyl-aminoindoles **2** [11].

**Scheme 1:**
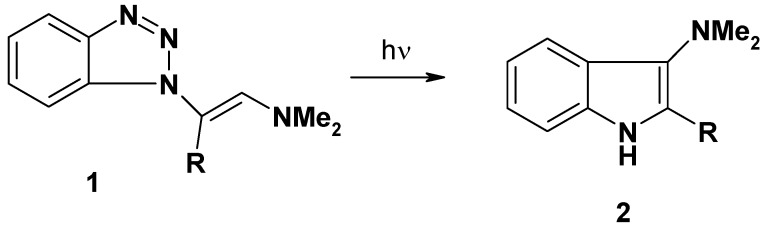
Photolysis of *N1*-vinylsubstituted benzotriazoles **1** into 2-acylindoles **2**.

**Figure 1:**
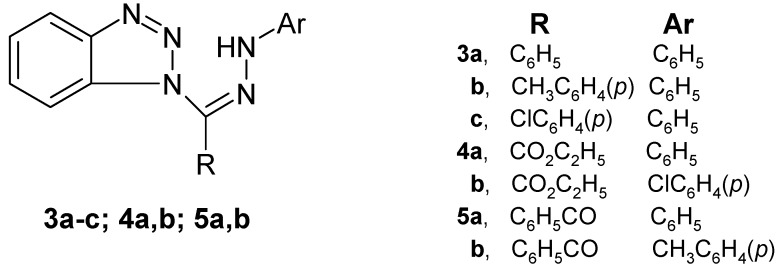
1-substituted benzotriazole arylhydrzones **3a–c**, **4a**,**b** and **5a**,**b**.

This investigation is now extended to include seven 1-substituted benzotriazole arylhydrzones **3a–c**, **4a**,**b** and **5a**,**b** (Figure 1).

## 2. Results and Discussion

Compounds **3a–c** and **5a**,**b** were obtained by the procedures described earlier and were fully characterized [12,13,14]. The UV spectra of these compounds display two absorption maxima in the region 243–392 nm wavelength regions.

**Scheme 2:**
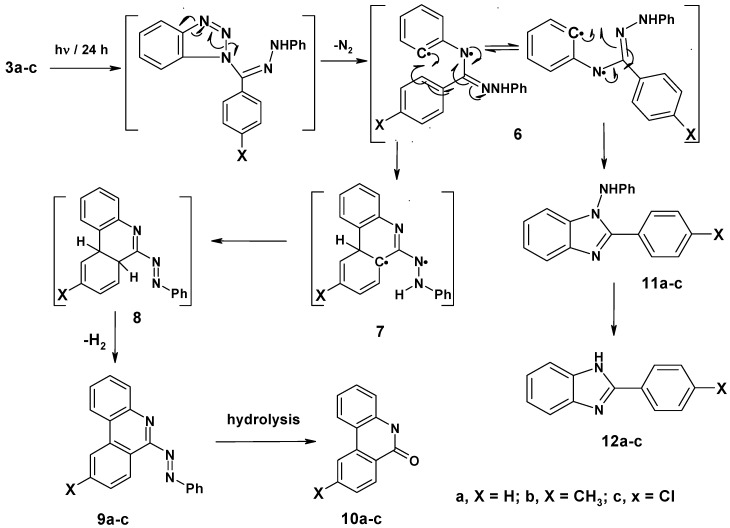
Mechanism of photolysis of *N*-(benzotriazol-1-yl-phenylmethylene)-*N′*-arylhydrazines **3a–c**.

**Figure 2:**
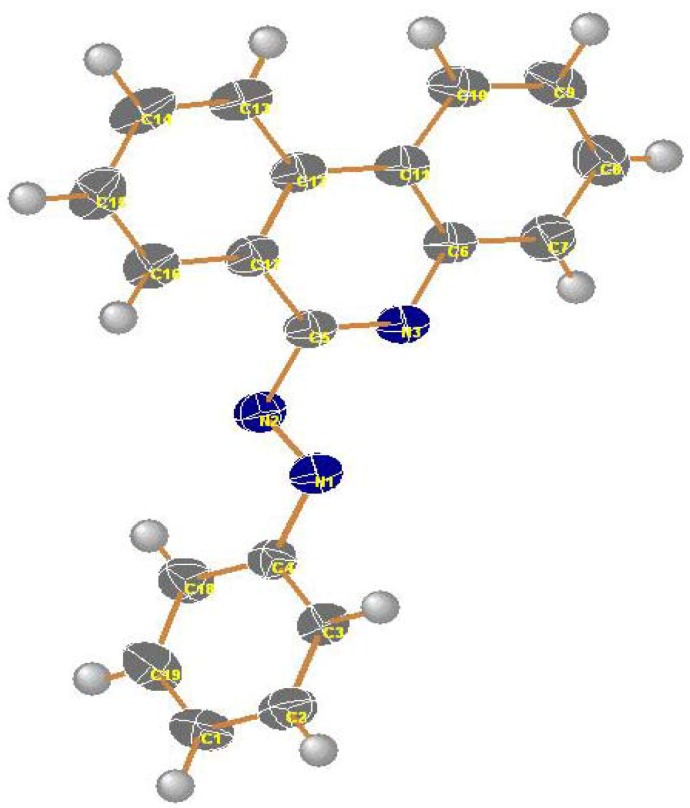
X-ray structure of compound **9a** (thermal ellipsoids).

Irradiation of compounds **3a–c** in a quartz tube with a 16 W low pressure mercury arc-lamp (254 nm) in acetonitrile for 24 hours at room temperature produced phenanthridin-6-yl-2-phenyldiazines **9a–c** (48–51%), phenanthridin-6(5*H*)-ones **10a–c** (15–18%), 1-anilino-2-arylbenzimidazoles **11a–c** (10–14%) and 2-aryl-1*H*-benzimidazoles **12a–c** (8–10%). The formation of these products can be explained by a mechanistic pathway presented in Scheme 2, involving initial photo-extrusion of N_2_ to form the corresponding diradical intermediate **6**, which cyclizes to diradical **7** and is then converted to **8**, and the latter is photooxidized to **9a–c**. Partial hydrolysis of **9a–c** in the reaction media produces **10a–c**. Concurrently, cyclization of the tautomer of diradical intermediate **6** affords compounds **11a–c** which are further converted to **12a–c**. To confirm that **9a–c** and **11a–c** are the likely precursors of **10a–c** and **12a–c** respectively, **9a** and **11a** were isolated and then subjected to irradiation for 24 hours. In each case the corresponding reaction products **10a** and **12a** were obtained in quantitative yield. The structures of all isolated products were confirmed based on their full ^1^H-NMR, ^13^C-NMR, and mass spectral data. Moreover, an X-ray crystal structure of compound **9a** was obtained (Figure 2 and Table 1).

**Table 1:** Selected bond lengths and bond angles for compound **9a**.

Bond	Bond lengths (Å)	Bond	Bond angles (Å)
N1-N2	1.2462 (13)	N1-N2-C5	112.58 (9)
N2-C5	1.4368 (14)	N3-C5-N2	117.90 (10)
N3-C6	1.3857 (15)	N2-C5-N17	116.62 (10)
N3-C5	1.2949 (15)	C5-N3-C6	117.99 (10)
C5-C17	1.4400 (16)	C6-C11-C12	118.27 (11)
N1-C4	1.4286 (14)	C10-C11-C12	124.11 (11)
C11-C12	1.4450 (18)	N3-C6-C11	122.68 (11)

**Scheme 3:**
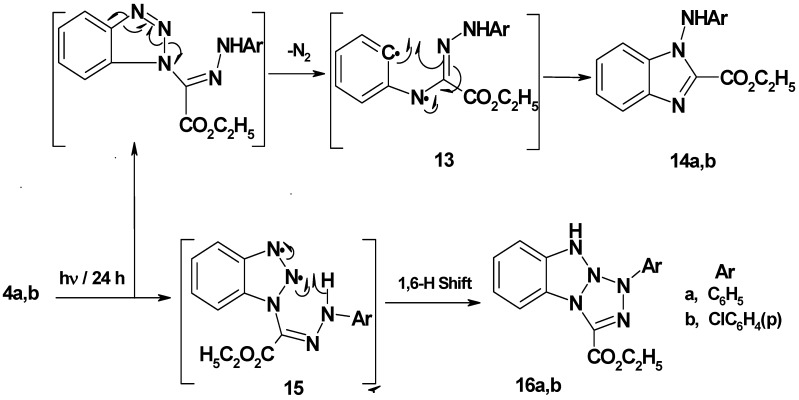
Mechanism of photolysis of benzotriazol-1-yl-(2-arylhydrazono)-acetic acid ethyl esters **4a**,**b**.

**Figure 3:**
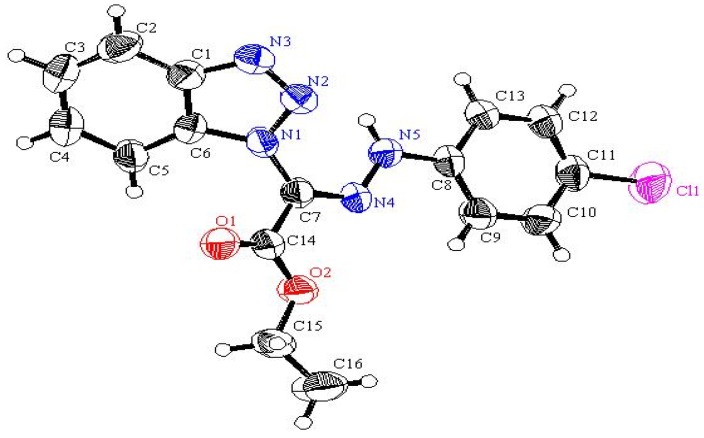
ORTEP drawing of **4b**.

**Figure 4:**
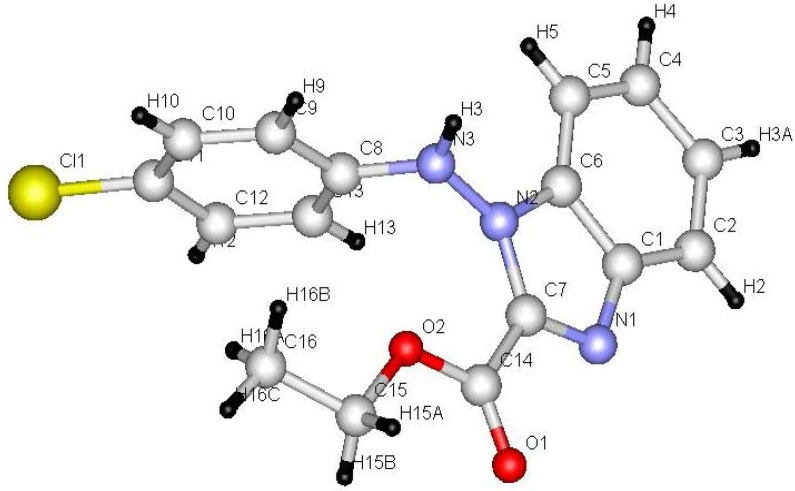
Ball and stick drawing of **14b**.

Benzotriazol-1-yl-(2-arylhydrazono) acetic acid ethyl esters **4a**,**b** were prepared in 75−77% yield by stirring 1,2,3-benzotriazole with ethyl 2-chloro-2-(2-arylhydrazono)acetate in DCM/TEA for 24 hours at room temperature. Irradiation of **4a**,**b** in acetonitrile for 24 hour afforded 1-arylamino-1*H*-benzimidazol-2-carboxylic acid ethyl esters **14a**,**b** (45−48% yield) and 1-aryl-1*H*,9*H*-benzo[4,5][1,2,3]triazolo[1,2-*a*]tetrazole-3-carboxylic acid ethyl esters **16a**,**b** (35−36% yield). It is assumed that initial N_2_ extrusion affords the biradical intermediate **13** which then cyclized into **14a**,**b**. On the other hand, formation of photoproducts **16a**,**b** may be explained by assuming a intramolecular 1,6-H shift (Scheme 3). Single crystal X-ray structure analysis (Figure 3 and Figure 4, Table 2) confirmed the structures of new compounds **4b** and **14b**.

**Table 2:** Selected bond lengths and bond angles for compounds **4b** and **14b**.

4b Bond	Bond lengths (Å)	Bond	Bond angles (°)	14b Bond	Bond lengths (Å)	Bond	Bond angles (°)
N1-C6	1.375 (6)	N1-C6-C1	104.8 (4)	N1-C1	1.380 (10)	C1-N1-C7	104.4 (6)
N1-C7	1.409 (6)	N1-C7-C14	117.1 (4)	N1-C7	1.318 (10)	N1-C7-N2	114.1 (7)
N4-C7	1.301 (6)	N1-C7-N4	125.3 (4)	N2-C7	1.375 (9)	N3-N2-C7	130.6 (6)
C7-C14	1.477 (7)	N1-N2-N3	108.5 (4)	N2-N3	1.372 (9)	N2-C6-C5	131.6 (8)
N5-C8	1.408 (6)	N2-N1-C7	120.3 (4)	N2-C6	1.405 (10)	N2-C7-C14	126.8 (7)
N4-N5	1.328 (6)	N5-C8-C13	118.2 (5)	C7-C14	1.495 (11)	N1-C1-C6	111.1 (7)
N3-C1	1.386 (7)	N3-C1-C6	109.2 (4)	N3-C8	1.409 (10)	O2-C14-C7	112.7 (7)

**Scheme 4:**
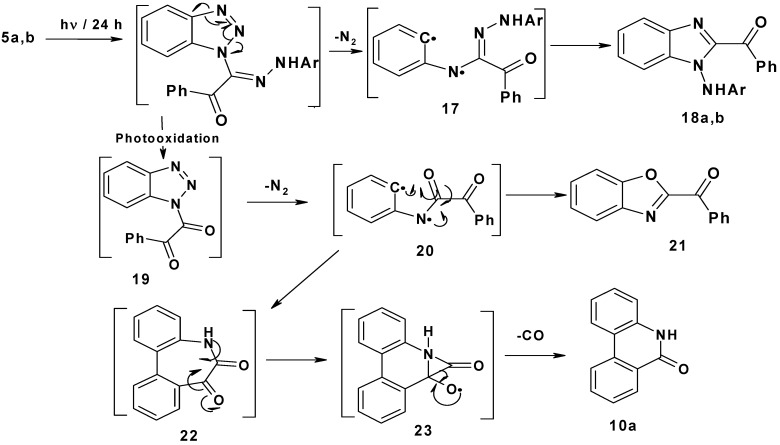
Mechanism of photolysis of 2-arylhydrazono-2-(benzotriazol-1-y)-1-phenylethanones **5a**,**b**.

Finally, irradiation of 2-arylhydrazono-2-(benzotriazol-1-yl)-1-phenylethanones **5a**,**b** afforded 1-arylamino-2-benzoylbenzimidazoles **18a**,**b** (25–30%), 2-benzoylbenzoxazole **21** (24–27%) and phenantheridin-6(5*H*)-one **10a** (15–17%). The suggested mechanism proposed for this photoreaction is shown in Scheme 4. Initial photo-extrusion of N_2_ forms the corresponding diradical intermediate **17**, followed by cyclization to yield **18a**,**b**. On the other hand, photooxidation of **5a**,**b** afforded 1-benzotriazole-2-phenylethan-1,2-dione **19**, which upon elimination of N_2_ formed diradical **20**, which either cyclizes to yield 2-benzoylbenzoxazole **21** in (24–27%) or cyclizes to **22**, which spontaneously loses CO through intermediate **23** to produce phenanthradin-6(5*H*)-one **10a** in 15–17% yield.

**Figure 5:**
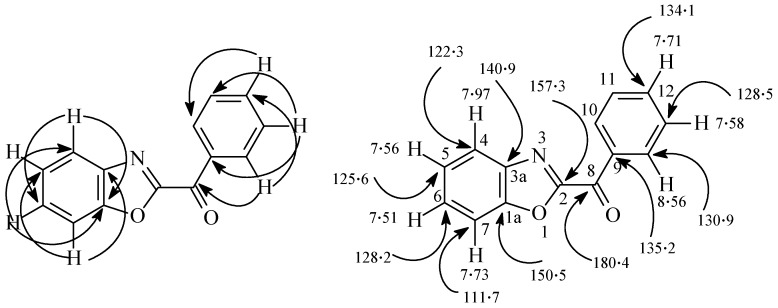
H-C Correlations in the HMBC 2-D experimental of compound **21**.

The structure of all new compounds were assigned by spectroscopic and analytical methods. The structure of **21** is readily assigned based on 2D-NMR results. The ^1^H- and ^13^C-NMR signal assignments and the H-C correlation from the HMBC 2-D experiments are displayed in Figure 5. Table 3 summarizes the absorption maxima (λ_max_) and the photoproducts of substrates **3a–c**, **4a**,**b** and **5a**,**b**. The fact that the substituents R affected the nature of the products much more than the substituted Ar may be attributed to the strong influence of the substituents on the formed biradicals.

**Table 3:** Photoproducts formed by irradiation of compounds **3a–c**, **4a**,**b** and **5a**,**b** and yield.

Comp	R	Ar	λ_max_	Photo-products and yields (%)							
				9	10	11	12	14	16	18	21
**3a**	C_6_H_5_	C_6_H_5_	244, 338	51	16	12	10	-	-	-	-
**3b**	CH_3_C_6_H_4_(*p*)	C_6_H_5_	247, 347	48	18	14	8	-	-	-	-
**3c**	ClC_6_H_4­_(*p*)	C_6_H_5_	243, 392	50	15	10	9	-	-	-	-
**4a**	CO_2_C_2_H_5_	C_6_H_5_	244, 370	-	-	-	-	48	36	-	-
**4b**	CO_2_C_2_H_5_	ClC_6_H_4_(*p*)	245, 355	-	-	-	-	45	35	-	-
**5a**	COC_6_H_5_	C_6_H_5_	253, 345	-	15	-	-	-	-	25	27
**5b**	COC_6_H_5_	CH_3_C_6_H_4_(*p*)	256, 379	-	17	-		-	-	30	24

## 3. Experimental

### 3.1. General

All melting points were recorded on a Gallenkamp apparatus. IR spectra were recorded using KBr pellets on a Perkin-Elmer System 2000 FT-IR spectrophotometer. ^1^H- and ^13^C-NMR spectra were recorded on Bruker DPX 400 MHz or Avance^II^ 600 MHz super-conducting NMR spectrometers with proton spectra measured at 400, 600 MHz and carbon spectra at 100 and 150 MHz, respectively. Mass spectra were measured on a VG Auto-spec-Q (high resolution, high performance, tri-sector GC/MS/MS) and with LCMS using Agilent 1100 series LC/MSD with an API-ES/APCI ionization mode. Microanalyses were performed on LECO CH NS-932 Elemental Analyzer. The UV/VIS absorption spectra were recorded using a Varian Cary 5 instrument in the wave length range 200–450 nm using dry clean quartz cuvette of 1.0 cm path length. X-Ray analysis were performed using a Rigaku Rapid II diffractometer.

### 3.2. Synthesis of Starting Compounds ***4a***,***b***

To a solution of 1,2,3-benzotriazole (3.57 g, 30 mmol), in DCM (50 mL), TEA (5 drops) and ethyl 2-chloro-2-(2-arylhydrazono)acetate (30 mmol) were added [15]. The mixture was stirred at room temperature for 24 hours, and then diluted with CH_2_Cl_2_ (150 mL), washed successively with dil. HCl (6 M, 20 mL), satd. aq. NaHCO_3_ (150 mL), and water and then dried over anhydrous Na_2_SO_4_. The solvent was then evaporated *in vacuo*, and the residue was crystallized from ethanol to give **4a**,**b**.

*Benzotriazol-1-yl-phenylhydrazono-acetic acid ethyl ester* (**4a**). Colorless crystals, yield 7.2 g (77%), m.p. 183–184 °C. MS: *m/z* (%) = 309 (M^+^, 15), 281 (35), 208 (80). IR (KBr, cm^−1^): 3060, 2982, 1683, 1598, 1540, 1501, 1414, 1312, 1235, 1187, 1019, 759. ^1^H-NMR (400 MHz, CDCl_3_): δ 9.69 (br, 1H, NH), 8.01 (d, 1H, *J =* 8.4 Hz), 7.59 (t, 1H, *J =* 8.0 Hz), 7.49 (d, 1H, *J* = 8.4 Hz), 7.43 (t, 1H, *J =* 8.0 Hz), 7.38–7.32 (m, 4H), 7.09 (t, 1H, *J =* 8.4 Hz), 4.41 (q, 2H, *J =* 7.0 Hz), 1.40 ppm (t, 3H, *J =* 7.0 Hz). ^13^C-NMR (100 MHz, CDCl_3_): δ 160.3, 144.9, 141.6, 132.5, 129.4, 128.9, 125.0, 123.8, 119.9, 118.5, 115.0, 111.2, 62.3, 14.3 ppm. Anal. Calcd. for C_16_H_15_N_5_O_2 _(309.3): C, 62.13; H, 4.89; N, 22.64. Found: C, 62.10; H, 4.80; N, 22.55.

*Benzotriazol-1-yl-p-chlorophenylhydrazono-acetic acid ethyl ester* (**4b**). Pale yellow crystals, yield 7.80 g (75%), m.p. 156–158 °C. MS: *m/z* (%) = 343 (M^+^, 10), 315 (30), 286 (35). IR (KBr, cm^−1^): 3094, 2986, 1686, 1571, 1547, 1489, 1401, 1282, 1235, 1172, 1148, 1092, 1061, 832. ^1^H-NMR (400 MHz, CDCl_3_): δ 9.77 (br, 1H, NH), 8.03 (d, 1H, *J* = 8.4 Hz), 7.60 (dt, 1H, *J =* 7.8, 1.2 Hz), 7.49 (d, 1H, *J =* 8.4 Hz), 7.44 (dt, 1H, *J =* 7.8, 1.2 Hz), 7.32 (d, 2H, *J =* 8.4 Hz), 7.24 (d, 2H, *J* = 8.4 Hz), 4.40 (q, 2H, *J =* 6.8 Hz), 1.24 ppm (t, 3H, *J =* 6.8 Hz). ^13^C-NMR (100 MHz, CDCl_3_): δ 160.1, 145.0, 140.4, 132.4, 129.5, 129.1, 128.7, 125.2, 120.0, 119.1, 116.2, 111.3, 62.4, 14.3 ppm. (HRMS = 343.0831, requires C_16_H_14_ClN_5_O_2_ 343.0830).

### 3.3. Irradiation Using a Low Pressure Mercury Arc-Lamp

Each of the substrates **3a–c**, **4a**,**b** and **5a**,**b** (10.0 mmol) was dissolved in acetonitrile (250 mL) in a number of quartz tubes (10 × 25 mL) and introduced to irradiate for 24 hours at room temperature (RT). The progress of each reaction was monitored by using TLC. The solvent was removed *in vacuo* and the resulting residue was subjected to column chromatography on silica gel using ethyl acetate/ petroleum ether (b.p. 60–80 °C) as the eluent to give the corresponding products.

*Phenanthridin-6-yl-2-phenyldiazine* (**9a**). Red crystals from ethanol, m.p. 158–160 °C. MS: *m/z* (%) = 283 (M^+^, 10), 254 (100), 178 (70). IR (KBr, cm^−1^): 3061, 3004, 2957, 1611, 1562, 1527, 1484, 1349, 1193, 925, 763. ^1^H-NMR (600 MHz, DMSO-d_6_): δ 8.98 (d, 1H, *J =* 7.8 Hz), 8.88 (d, 1H, *J =* 8.4 Hz), 8.55 (d, 1H, *J =* 8.0 Hz), 8.18 (d, 1H, *J =* 8.0 Hz), 8.15 (dd, 2H, *J =* 7.8, 1.2 Hz), 8.07 (t, 1H, *J =* 7.6 Hz), 7.88 (t, 1H, *J =* 7.8 Hz), 7.84 (t, 1H, *J =* 7.6 Hz), 7.81 (t, 1H, *J =* 7.6 Hz), 7.73–7.70 ppm (m, 3H). ^13^C-NMR (150 MHz, DMSO-d_6_): δ 159.6, 152.5, 142.4, 133.8, 133.0, 131.9, 130.5, 129.7, 129.6, 128.3, 127.9, 125.7, 124.5, 123.4, 123.0, 122.9 122.1 ppm. Anal. Calc. for C_19_H_13_N_3_ (283.3): C, 80.54; H, 4.62; N, 14.83. Found: C, 80.50; H, 4.60; N, 14.79.

*9-Methylphenanthridin-6-yl-2-phenyldiazine* (**9b**). Red crystals from ethanol, m.p. 140–142 °C. MS: *m/z* (%) = 297 (M^+^, 10), 268 (100), 192 (40). IR (KBr, cm^−1^): 3056, 2918, 1617, 1509, 1483, 1373, 1308, 1189, 1022, 822, 760. ^1^H-NMR (400 MHz, CDCl_3_): δ 8.62 (dd, 2H, *J =* 8.4, 2.0 Hz), 8.50 (s, 1H), 8.31 (d, 1H, *J =* 8.0 Hz), 8.21 (dd, 2H, *J* = 8.4, 2.0 Hz), 7.76 (t, 1H, *J =* 7.8 Hz), 7.70 (t, 1H, *J =* 7.8 Hz), 7.63–7.58 (m, 4H), 2.70 ppm (s, 3H, CH_3_). ^13^C-NMR (150 MHz, DMSO-d_6_): δ 159.5, 153.3, 143.3, 141.9, 134.9, 132.4, 131.6, 129.4, 129.2, 129.1, 127.4, 126.3, 125.0, 124.0, 122.1, 121.9, 121.7, 22.5 ppm. Anal. Calc. for C_20_H_15_N_3_ (297.4): C, 80.78; H, 5.08; N, 14.13. Found: C, 80.70; H, 5.00; N, 14.10.

*9-Chlorophenanthridin-6-yl-2-phenyldiazine* (**9c**). Red crystals from ethanol, m.p. 136–138 °C. MS: *m/z* (%) = 319 (M^+2^, 10), 317 (M^+^, 20), 268 (100). IR (KBr, cm^−1^): 3062, 3007, 2957, 1605, 1512, 1486, 1380, 1309, 1143, 1015, 910, 761. ^1^H-NMR (400 MHz, CDCl_3_): δ 8.70 (d, 1H, *J =* 8.4 Hz), 8.66 (s, 1H), 8.53 (d, 1H, *J =* 8.0 Hz), 8.33 (d, 1H, *J =* 7.8 Hz), 8.19 (dd, 2H, *J =* 7.8, 1.2 Hz), 7.81 (t, 1H, *J =* 7.8 Hz), 7.73 (t, 1H, *J* = 8.0 Hz), 7.70 (dd, 1H, *J* = 8.0, 1.2 Hz), 7.62-7.58 ppm (m, 3H). ^13^C-NMR (100 MHz, CDCl_3_): δ 158.9, 153.3, 143.6, 138.0, 135.9, 132.7, 131.7, 129.9, 129.2, 128.3, 128.2, 127.9, 127.3, 124.1, 124.0, 122.2, 122.0 ppm. Anal. Calc. for C_19_H_12_ClN_3_ (317.8): C, 71.81; H, 3.81; N, 13.22. Found: C, 71.75; H, 3.80; N, 13.17.

*Phenanthridin-6(5H)-one* (**10a**). White crystals, mp. 289–290 °C (lit. [13] m.p. 290–292 °C). MS: *m/z* (%) = 195 (M^+^, 100), 167 (20), 139 (15). ^1^H-NMR (400 MHz, DMSO-d_6_): δ 11.70 (br, 1H, NH), 8.52 (d, 1H, *J =* 8.0 Hz), 8.40 (d, 1H, *J* = 8.0 Hz), 8.33 (dd, 1H, *J =* 8.0, 1.2 Hz), 7.82 (dt, 1H, *J =* 8.0, 1.2 Hz), 7.65 (t, 1H, *J* = 8.0 Hz), 7.49 (dt, 1H, *J =* 8.0, 1.4 Hz), 7.36 (dd, 1H, *J* = 8.0, 1.4 Hz), 7.27 ppm (dt, 1H, *J* = 8.0, 1.4 Hz). ^13^C-NMR (100 MHz, DMSO-d_6_): δ 160.9, 136.6, 134.3, 132.9, 129.6, 128.0, 127.5, 125.7, 123.3, 122.7, 122.3, 117.6, 116.1 ppm.

*9-Methylphenanthridin-6(5H)-one* (**10b**). Colorless crystals, m.p. 251–253 °C (lit. [16] m.p. 250–251 °C). MS: *m/z* (%) = 209 (M^+^, 100), 180 (25). ^1^H-NMR (600 MHz, DMSO-d_6_): δ 11.58 (br, 1H, NH), 8.36 (d, 1H, *J* = 7.8 Hz), 8.32 (s, 1H), 8.19 (d, 1H, *J =* 8.0 Hz), 7.46 (dt, 2H, *J* = 8.4,1.6 Hz), 7.34 (dd, 1H, *J =* 8.0,1.4 Hz), 7.25 (dt, 1H, *J* = 8.4,1.2 Hz), 2.51 ppm (s, 3H, CH_3_). ^13^C-NMR (150 MHz, DMSO-d_6_): δ 160.8, 143.0, 136.7, 134.2, 129.4, 129.1, 127.5, 123.4, 123.2, 122.5, 122.1, 117.5, 116.1, 21.5 ppm.

*9-Chlorophenanthridin-6(5H)-one* (**10c**). Colorless crystals, m.p. 268–270 °C. LCMS: *m/z* = 232 (M + 3), 230 (M + 1). ^1^H NMR (400 MHz, CDCl_3_): δ 11.53 (br, 1H, NH), 8.43 (d, 1H, *J =* 8.0 Hz), 8.25 (d, 1H, *J =* 8.0 Hz), 8.14 (d, 1H, *J =* 8.4 Hz), 7.80 (t, 1H, *J =* 7.8 Hz), 7.64 (t, 1H, *J =* 7.8 Hz), 7.45 (d, 1H, *J =* 8.0 Hz), 7.38 ppm (t, 1H, *J =* 7.8 Hz) [13].

*1-Anilino-2-phenylbenzimidazole* (**11a**). Colorless crystals, m.p. 211–212 °C (lit. [14] m.p. 210–212 °C). LCMS: *m/z* = 286 (M + 1). ^1^H-NMR (400 MHz, CDCl_3_): δ 8.07 (m, 2H), 7.86 (d, 1H, *J =* 8.0 Hz), 7.46–7.43 (m, 3H), 7.34 (t, 1H, *J =* 7.8 Hz), 7.32–7.24 (m, 3H), 7.21 (t, 1H, *J =* 8.4 Hz), 7.00 (t, 1H, *J =* 7.6 Hz), 6.81 (br, 1H, NH), 7.72 ppm (d, 2H,*J =* 7.8 Hz).

*1-Anilino-2-p-tolylbenzimidazole* (**11b**). Colorless crystals, m.p. 233–235 °C (lit. [14] m.p. 234–236 °C). LCMS: *m/z* = 300 (M + 1). ^1^H-NMR (CDCl_3_): δ 8.05 (d, 2H, *J =* 8.0 Hz), 7.82 (d, 1H, *J =* 8.0 Hz), 7.44 (d, 2H, *J =* 8.4 Hz), 7.33 (t, 1H, *J =* 7.8 Hz), 7.28 (m, 2H), 7.17 (d, 1H, *J =* 7.8 Hz), 7.02 (t, 2H, *J =* 7.8 Hz), 6.81 (br, 1H), 6.67 (d, 2H, *J =* 8.0 Hz), 2.40 ppm (s, 3H, CH_3_).

*1-Anilino-2-p-chlorophenylbenzimidazole* (**11c**). Colorless crystals, m.p. 230–233 °C (lit. [14] m.p. 232–234 °C). LCMS: *m/z* = 321 (M + 2), 320 (M + 1). ^1^H-NMR (400 MHz, CDCl_3_): δ 8.05 (dd, 2H, *J =* 8.4, 1.6 Hz), 7.86 (d, 1H, *J =* 8.4 Hz), 7.43 (dd, 2H, *J =* 8.4, 1.6 Hz), 7.35 (t, 1H, *J =* 8.0 Hz), 7.30–7.25 (m, 3H), 7.17 (d, 1H, *J =* 8.0 Hz), 7.02 (t, 1H, *J =* 7.8 Hz), 6.81 (br, 1H, NH), 6.67 ppm (d, 2H, *J =* 8.0 Hz).

*2-Phenyl-1H-benzimidazole* (**12a**). Colorless crystals, m.p. 290–292 °C (lit. [14] m.p. 289–290 °C). LCMS: *m/z* = 195 (M + 1). ^1^H-NMR (400 MHz, CDCl_3_): δ 8.12 (m, 2H), 7.68 (m, 2H), 7.45 (m, 3H), 7.29 ppm (m, 3H, *J* 7.8 Hz).

*2-p-Tolyl-1H-benzimidazole* (**12b**). Colorless crystals, m.p. 271–272 °C (lit. [14] m.p. 269–272 °C). LCMS: *m/z* = 209 (M + 1). ^1^H-NMR (400 MHz, DMSO-d_6_): δ 12.78 (br, 1H, NH), 8.06 (d, 2H, *J =* 8.0 Hz), 7.56 (m, 2H), 7.32 (d, 2H, *J =* 8.0 Hz), 7.16 (m, 2H), 2.33 ppm (s, 3H, CH_3_).

*2-p-Chlorophenyl-1H-benzimidazole* (**12c**). Colorless crystals, m.p. 291–292 °C (lit. [14] m.p. 289–291 °C). LCMS: *m/z* = 231 (M + 3), 229 (M + 1). ^1^H-NMR (400 MHz, DMSO-d_6_): δ 12.98 (br, 1H, NH), 8.18 (d, 2H, *J =* 8.4 Hz), 7.58 (d, 2H, *J =* 8.4 Hz), 7.56 (m, 2H), 7.18 ppm (m, 2H).

*1-Anilino-1H-benzimidazole-2-carboxylic acid ethyl ester* (**14a**). Colorless crystals from ethanol, m.p. 142–144 °C. MS: *m/z* (%) = 281 (M^+^, 70), 253 (35), 208 (80). IR (KBr, cm^−1^): 3244, 3054, 2973, 1683, 1600, 1556, 1490, 1392, 1240, 1157, 1053, 734. ^1^H-NMR (600 MHz, CDCl_3_): δ 7.95 (d, 1H, *J* = 8.4 Hz), 7.76 (br, 1H, NH), 7.56 (dd, 1H, *J* = 8.4, 1.2 Hz), 7.45 (d, 1H, *J* = 8.4 Hz), 7.36 (dt, 1H, *J* = 7.8, 1.4 Hz), 7.12 (t, 2H, *J* = 8.4 Hz), 6.98 (t, 1H, *J* = 7.8 Hz), 6.54 (d, 2H, *J* = 8.0 Hz), 4.43 (q, 2H, *J* = 6.8 Hz), 1.42 ppm (t, 3H, *J* = 6.8 Hz). ^13^C-NMR (150 MHz, CDCl_3_): δ 159.8, 144.7, 141.2, 132.1, 128.9, 128.4, 124.5, 123.3, 119.6, 118.1, 114.5, 110.7, 61.7, 13.7 ppm. Anal. Calcd. for C_16_H_15_N_3_O_2 _(281.3): C, 68.30; H, 5.37; N, 14.94. Found: C, 68.25; H, 5.35; N, 14.89.

*1-p-Chloroanilino-1H-benzimidazole-2-carboxylic acid ethyl ester* (**14b**). Colorless crystals from ethanol, m.p. 146–148 °C. MS: *m/z* (%) = 315 (M^+^, 100), 242 (65), 149 (80). IR (KBr, cm^−1^): 3224, 3032, 2979, 1723, 1599, 824. ^1^H-NMR (600 MHz, CDCl_3_): δ 7.97 (d, 1H, *J* = 8.4 Hz), 7.76 (br, 1H, NH), 7.56 (dd, 1H, *J* = 8.4,1.4 Hz), 7.53 (dt, 1H, *J* = 8.4, 1.4 Hz), 7.45 (dt, 1H, *J* = 7.8, 1.4 Hz), 7.18 (d, 2H, *J* = 8.4 Hz), 6.48 (d, 2H, *J* = 8.4 Hz), 4.43 (q, 2H, *J* = 6.8 Hz), 1.42 ppm (t, 3H, *J* = 6.8 Hz). ^13^C-NMR (150 MHz, CDCl_3_): δ 159.2, 145.6, 138.9, 138.8, 135.5, 129.5, 127.6, 126.9, 124.7, 122.1, 115.1, 110.8, 62.7, 14.1 ppm. (HRMS = 315.0769; requires 315.0768).

*1-Phenyl-1H,9H-benzo[4,5][1,2,3]triazolo[1,2-a]tetrazole-3-carboxylic acid ethyl ester* (**16a**). Pale yellow crystals from ethanol, m.p. 152–154 °C. MS: *m/z* (%) = 309 (M^+^, 25), 295 (10), 281 (50). IR (KBr, cm^−1^): 3036, 2982, 1683, 1598, 1540, 1501, 1458, 1312, 1235, 1187, 1063, 1019, 759. ^1^H-NMR (CDCl_3_): δ 12.44 (s, 1H, NH), 8.16 (d, 1H, *J* = 8.4 Hz), 7.60–7.54 (m, 2H), 7.45 (tt, 1H, *J* = 8.4, 1.6 Hz), 7.37 (t, 2H, *J =* 8.0 Hz), 7.28 (d, 2H, *J =* 8.4 Hz), 7.12 (t, 1H, *J =* 7.8 Hz), 4.31 (q, 2H, *J =* 7.2 Hz), 1.22 ppm (t, 3H, *J =* 7.2 Hz). ^13^C-NMR (100 MHz, CDCl_3_): δ 161.9, 145.8, 142.3, 134.6, 130.1, 130.0, 128.83, 124.80, 124.76, 120.6, 115.5, 110.8, 62.9, 14.5 ppm. Anal. Calcd. for C_16_H_15_N_5_O_2_ (309.3): C, 62.13; H, 4.89; N, 22.64. Found: C, 62.07; H, 4.82; N, 22.65.

*1-p-Chlorophenyl-1H,9H-benzo[4,5][1,2,3]triazolo[1,2-a]tetrazole-3-carboxylic acid ethyl ester* (**16b**). Yellow crystals from ethanol, m.p. 156–158 °C. MS: *m/z* (%) = 343 (M^+^, 15), 315 (30), 126 (100). IR (KBr, cm^−1^): 3094, 2986, 1680, 1571, 1547, 1489, 1401, 1282, 1235, 1172, 1148, 1062, 832. ^1^H-NMR (400 MHz, CDCl_3_): δ 12.43 (s, 1H, NH), 8.15 (d, 1H, *J* = 8.0 Hz), 7.58–7.55 (m, 2H), 7.48–7.44 (m, 1H), 7.32 (d, 2H, *J* = 8.4 Hz), 7.22 (d, 2H, *J =* 8.4 Hz), 4.31 (q, 2H, *J =* 7.2 Hz), 1.23 ppm (t, 3H, *J =* 7.2 Hz). ^13^C-NMR (100 MHz, CDCl_3_): δ 161.3, 145.2, 140.3, 133.9, 129.6, 129.2, 128.3, 124.2, 120.1, 119.2, 116.1, 110.1, 62.4, 13.9 ppm. (HRMS = 343.0830; requires C_16_H_14_ClN_5_O_2_ 343.0831).

*1-Anilino-2-benzoylbenzimidazole* (**18a**). Yellow crystals, m.p. 216–218. °C. MS: *m/z* (%) = 313 (M^+^, 60), 279 (20), 167 (40), 149 (100). ^1^H-NMR (400 MHz, CDCl_3_): δ 8.33 (dd, 2H, *J* = 8.4, 1.6 Hz), 8.12 (br, 1H, NH), 7.96 (d, 1H, *J* = 8.0 Hz), 7.70 (dt, 1H, *J* = 8.4, 1.6 Hz), 7.60 (dt, 1H, *J* = 8.0, 1.4 Hz), 7.53–7.42 (m, 3H), 7.34 (t, 1H, *J =* 7.8 Hz), 7.16 (t, 2H, *J* = 8.0 Hz), 6.90 (t, 1H, *J* = 8.2 Hz), 6.54 ppm (d, 2H, *J* = 7.8 Hz). ^13^C-NMR (100 MHz, CDCl_3_): δ 185.7, 147.2, 144.7, 139.1, 135.7, 134.0, 131.2, 129.4, 128.3, 127.6, 126.9, 124.4, 122.4, 122.3, 113.8, 111.1 ppm. (HRMS = 313.1209; requires C_20_H_15_N_3_O 313.1207).

*2-Benzoyl-1-p-toluidinobenzimidazole* (**18b**). Pale yellow crystals, m.p. 226–228 °C. MS: *m/z* (%) = 327 (M^+^, 100), 223 (40), 195 (100). IR (KBr, cm^−1^): 3331, 3061, 1648, 730. ^1^H-NMR (400 MHz, CDCl_3_): δ 8.34 (dd, 2H, *J =* 8.4, 1.6 Hz), 8.12 (br, 1H, NH), 7.99 (d, 1H, *J =* 8.0 Hz), 7.73 (d, 1H, *J =* 8.4 Hz), 7.64–7.50 (m, 3H), 7.34 (t, 1H, *J =* 7.8 H), 7.06 (t, 1H, *J =* 8.0 Hz), 6.99 (d, 2H, *J =* 8.8 Hz), 6.49 (d, 2H, *J =* 8.8 Hz), 2.22 ppm (s, 3H, CH_3_). ^13^C-NMR (100 MHz, CDCl_3_): δ 185.4, 144.5, 137.8, 136.2, 134.6, 132.1, 131.5, 130.0, 129.8, 129.3, 128.9, 127.4, 126.1, 122.3, 114.1, 111.5, 20.6 ppm. (HRMS = 327.1366, requires C_21_H_17_N_3_O 327.1366).

*2-Benzoylbenzoxazole* (**21**). Colorless crystals from ethanol, m.p. 136–138 (lit. [17] m.p. 139 °C). MS: *m/z* (%) = 223 (M^+^, 75), 195 (40), 105 (100). ^1^H-NMR (600 MHz, CDCl_3_): δ 8.56 (dd, 2H, *J =* 8.4, 1.6 Hz), 7.97 (d, 1H, *J =* 8.4 Hz), 7.73 (d, 1H, *J =* 8.4 Hz), 7.71 (t, 1H, *J =* 8.0 Hz), 7.58 (t, 2H, *J* = 7.8 Hz), 7.56 (t, 1H, *J* = 7.8 Hz), 7.51 ppm (t, 1H, *J =* 7.8 Hz). ^13^C-NMR (150 MHz, CDCl_3_): δ 180.4 (C), 157.3 (C), 150.5 (C), 140.9 (C), 135.2 (C), 134.1 (CH), 130.9 (2CH), 128.5 (2CH), 128.2 (CH), 125.6 (CH), 122.3 (CH), 111.7 (CH) ppm. Anal. Calcd. for C_14_H_9_NO_2_ (223.3): C, 75.33; H, 4.06; N, 6.27. Found: C, 75.23; H, 4.05; N, 6.29.

## 4. Conclusions

The present study offers a new route for the synthesis of some new heterocyclic phenanthridin-6-yl-2-phenyldiazines. Some of these photoproducts **10a–c** have been shown to have efficient compelexation properties with transition metals and exhibit interesting photo-emission and fluorescence properties [18,19]. It also shows that 1-substituted benzotriazole arylhydrazones behave photochemically in a different manner than in flash vacuum pyrolysis (FVP) or static pyrolysis (STP) reactions [12,13,14].
